# Lanthanide-Doped Upconversion Nanoparticles for Super-Resolution Microscopy

**DOI:** 10.3389/fchem.2020.619377

**Published:** 2021-01-15

**Authors:** Hao Dong, Ling-Dong Sun, Chun-Hua Yan

**Affiliations:** ^1^Beijing National Laboratory for Molecular Sciences, State Key Laboratory of Rare Earth Materials Chemistry and Applications, PKU-HKU Joint Laboratory in Rare Earth Materials and Bioinorganic Chemistry, College of Chemistry and Molecular Engineering, Peking University, Beijing, China; ^2^College of Chemistry and Chemical Engineering, Lanzhou University, Lanzhou, China

**Keywords:** upconversion nanoparticle, super-resolution microscopy, lanthanide, STED, multiphoton imaging

## Abstract

Super-resolution microscopy offers a non-invasive and real-time tool for probing the subcellular structures and activities on nanometer precision. Exploring adequate luminescent probes is a great concern for acquiring higher-resolution image. Benefiting from the atomic-like transitions among real energy levels, lanthanide-doped upconversion nanoparticles are featured by unique optical properties including excellent photostability, large anti-Stokes shifts, multicolor narrowband emissions, tunable emission lifetimes, etc. The past few years have witnessed the development of upconversion nanoparticles as probes for super-resolution imaging studies. To date, the optimal resolution reached 28 nm (λ/36) for single nanoparticles and 82 nm (λ/12) for cytoskeleton structures with upconversion nanoparticles. Compared with conventional probes such as organic dyes and quantum dots, upconversion nanoparticle-related super-resolution microscopy is still in the preliminary stage, and both opportunities and challenges exist. In this perspective article, we summarized the recent advances of upconversion nanoparticles for super-resolution microscopy and projected the future directions of this emerging field. This perspective article should be enlightening for designing efficient upconversion nanoprobes for super-resolution imaging and promote the development of upconversion nanoprobes for cell biology applications.

## Introduction

Optical microscopy that can visualize the morphological and physiological details of biological samples with high sensitivity as well as non-invasive and real-time capability has become an important part for diagnosis (Biffi et al., 2013; Chen et al., 2013; Tang et al., 2013). Nonetheless, the Abbe's diffraction limit constrains the imaging resolution in hundreds of nanometers (*ca*. λ/2) and makes it impossible to access the subcellular organelles' detection (Abbe, 1873). With the development of super-resolution microscopy since the 1990's, the optical diffraction limit has been circumvented or even broken, leading to the unprecedented observation of nanoscale subcellular structures and dynamics of cells and tissues (Hell and Wichmann, 1994; Betzig et al., 2006; Rust et al., 2006). In super-resolution imaging, luminescent probes are key components for labeling and disclosing the structure and activities of target molecules in nanometer precision. An ideal probe should have adequate brightness, excellent photostability, good biocompatibility, etc (Sahl et al., 2017). In recent years, lanthanide-doped upconversion nanoparticles (UCNPs) that can transduce near-infrared (NIR) photons to visible emissions have been emerging as a new kind of probe for super-resolution microscopy (Dong et al., 2020). The atomic-like transitions among real 4*f* energy levels endow UCNPs with unique optical properties, including vigorous resistance to photobleaching and photoblinking, efficient anti-Stokes emission efficiency, and being free of autofluorescence interference, which are not inherent in conventional probes such as organic dyes and quantum dots (Chan et al., 2015; Dong et al., 2015a; Zheng et al., 2015). UCNPs with high monodispersity, uniform shape, and phase structure can be produced by well-developed approaches such as high-temperature thermal decomposition (Mai et al., 2006) and coprecipitation (Li and Zhang, 2008). The size of UCNPs can be controlled from sub-5 nm to super-100 nm with narrow distribution. The physical characters including size, hydrodynamic diameter, and concentration can be obtained with transmission electron microscopy (TEM), dynamic light scattering (DLS), and inductively coupled plasma-atomic emission spectroscopy (ICP-AES), respectively. Moreover, with proper surface modification (Muhr et al., 2014), UCNPs can well be internalized by cells through endocytosis and show no obvious toxic effect on cell proliferation and viability (Gnach et al., 2015). These merits make UCNPs greatly promising for long-term and real-time observations.

To date, UCNPs have been implanted in diverse super-resolution imaging techniques, including ion beam excitation (Mi et al., 2015), stimulated emission depletion (STED) microscopy (Kolesov et al., 2011), fluorescence emission difference (FED) microscopy (Wu et al., 2017), and structured illumination microscopy (SIM) (Liu et al., 2020). The spatial resolution of 28 nm (λ/36) and 82 nm (λ/12) has been achieved for single UCNPs (Liu et al., 2017) and UCNPs-labeled cytoskeleton proteins (Zhan et al., 2017), respectively, which is greatly improved than the λ/2 resolution in conventional confocal imaging. Despite these achievements, great challenges still exist for the super-resolution imaging with UCNPs. On one hand, the 4*f* intra-configurational transitions of lanthanides are shielded by 5*s*^2^5*p*^6^ shells; thus, the energy level position as well as the excitation/emission wavelengths are almost invariable (Dong et al., 2015b). This actually poses a challenge for tuning the luminescence properties of UCNPs, which are not as flexible as that of organic dyes and quantum dots. On the other hand, the stimulated emission cross-section of UCNPs is quite small because of the parity-forbidden nature (Sun et al., 2019), which makes it difficult to achieve stimulated emission for STED microscopy. In this perspective article, we firstly summarized the recent advances in UCNPs-based super-resolution microscopy and then discussed the future directions in aspects of UCNPs design and cellular biological applications.

## Recent Advances in UCNPs-Based Super-Resolution Imaging

### UCNPs in Ion Beam Excitation

**Figure 1:**
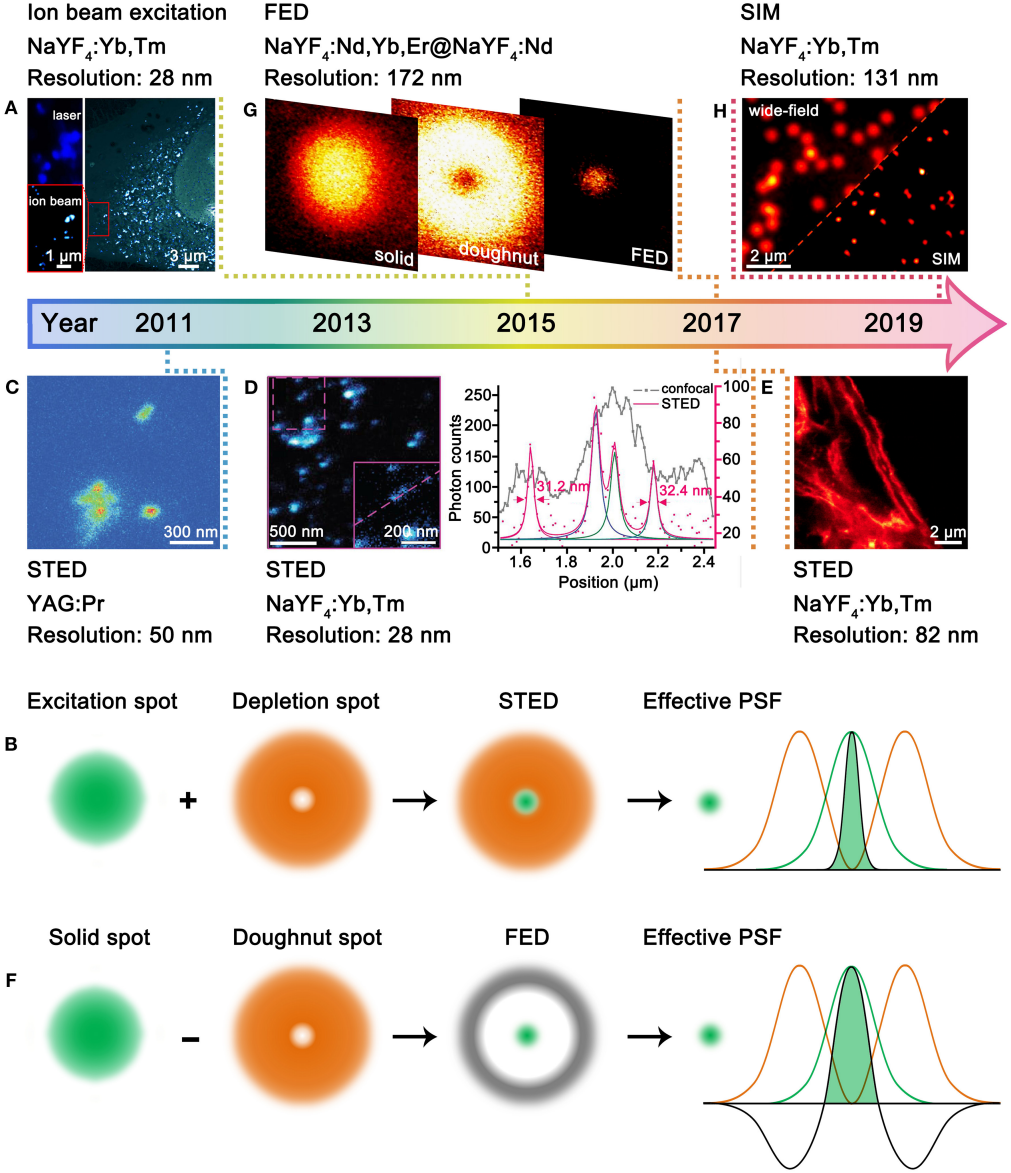
Development of UCNPs for super-resolution microscopy. **(A)** Comparative photoluminescence imaging under 980-nm excitation (top left) and ionoluminescence imaging under helium ion beam excitation (bottom left) of NaYF_4_:Yb,Tm UCNPs in a HeLa cell (right). Reproduced with permission from Mi et al. (2015). Copyright 2015 Springer Nature Publishing Group. **(B)** Schematic illustration for STED microscopy. **(C)** STED imaging of YAG:Pr nanoparticle clusters. Reproduced with permission from Kolesov et al. (2011). Copyright 2011 American Physical Society. **(D)** STED imaging of *ca*. 13 nm NaYF_4_:Yb,Tm UCNPs (left) and the intensity profiles along the dashed line (right). Reproduced with permission from Liu et al. (2017). Copyright 2017 Springer Nature Publishing Group. **(E)** STED imaging of cytoskeleton structures and desmin proteins in HeLa cancer cells with NaYF_4_:Yb,Tm UCNPs. Reproduced with permission from Zhan et al. (2017). Copyright 2017 Springer Nature Publishing Group. **(F)** Schematic illustration for FED microscopy. **(G)** FED imaging of a single NaYF_4_:Nd,Yb,Er@NaYF_4_:Nd UCNPs. Reproduced with permission from Wu et al. (2017). Copyright 2017 The Optical Society. **(H)** Comparative wide-field and SIM imaging of NaYF_4_:Yb,Tm UCNPs. Reproduced with permission from Liu et al. (2020). Copyright 2020 American Chemical Society.

According to Abbe's diffraction limit, the spatial resolution is proportional to the excitation wavelength. Therefore, excitation with charged particles such as electron or ion beams that have much shorter de Broglie wavelength can accomplish much better resolution than that with usual UV-vis-NIR lasers (Prigozhin et al., 2019). This approach has no specific requirements for the composition of UCNPs, yet more sensitizers are beneficial for the increased absorption of excitation energy. Bettiol et al. presented a paradigmatic example by using MeV focused helium ions to excite NaYF_4_:Yb,Tm UCNPs (Figure 1A), in which Yb^3+^ and Tm^3+^ ions can directly harvest the energy of helium ions and stably generate upconversion emissions over hours period (Mi et al., 2015). Compared with 980-nm laser excitation, the helium ion beam excitation enabled greatly improved resolution from 253 to 28 nm. Moreover, an areal density map of the distribution of individual UCNPs within a whole HeLa cell as well as the 3D cellular structure can be revealed at ultrahigh spatial resolution. Although the ion beam excitation can generate super-resolution imaging, the special imaging setups, especially the high-energy ion beams, are not easily accessible. Moreover, the high-energy ion beam may induce irreversible damage to living biological samples, which thus should have certain limitations in practical use.

### UCNPs in Stimulated Emission Depletion Microscopy

In the 1990's, STED microscopy was theoretically proposed (Hell and Wichmann, 1994) and experimentally validated (Klar and Hell, 1999) to break the diffraction barrier by reducing the point spread function (PSF) with two concentrical laser beams, namely, an excitation laser and a depletion laser (Figure 1B). The advantage of STED microscopy lies in the direct acquisition of a super-resolved image without image post-processing. A qualified STED probe should exhibit fluorescence and stimulated emission under excitations of excitation laser and depletion laser, respectively. In this way, the undesired fluorescence can be erased, leading to shrunk effective PSF. The excitation-correlated emission property is easy to be implemented for organic dyes (Willig et al., 2006) and quantum dots (Hanne et al., 2015), which however is a daunting challenge for lanthanide-doped UCNPs. This can be ascribed to the two following aspects: (I) Stimulated emission is quite difficult to be generated from single UCNPs because of the small stimulated emission cross-section. (II) The co-illumination of excitation and depletion lasers is much likely to match the abundant energy levels of lanthanides, which is deleterious to erasing process. To circumvent the two aforementioned challenges, the UCNPs for STED microscopy are designed to show depleted upconversion emissions through cross-relaxations under the co-illumination of excitation and depletion lasers. Therefore, there are strict requirements for the composition and excitation conditions of UCNPs in STED microscopy, and only few types of UCNPs have been developed.

In 2011, Kolesov et al. employed UCNPs for STED microscopy for the first time (Figure 1C) (Kolesov et al., 2011). The upconversion emission properties of YAG:Pr nanoparticles were modulated by three lasers, including pulsed excitation, continuous depletion, and pulsed readout. The accomplishment of super-resolution was based on the longer lifetime of the intermediate state (*ca*. 150–200 μs) than that of the excited state (*ca*. 18 ns). Although the resolution was improved from *ca*. 400 nm to *ca*. 50 nm, the imaging wavelength was concentrated at the UV region (300–450 nm), which is deleterious to detection and bioapplications (Idris et al., 2015). In 2017, Jin et al. made a significant promotion of upconversion STED microscopy with NaYF_4_:Yb,Tm UCNPs (Figure 1D) (Liu et al., 2017). A 980-nm excitation laser (*ca*. 10^4^-10^5^ W/cm^2^) and an 808-nm depletion laser (*ca*. 10^6^-10^7^ W/cm^2^) were used to activate and quench the blue upconversion emissions of Tm^3+^, respectively, which is much easier to operate. Heavy doping of Tm^3+^ was found crucial to depopulate the blue-emitting energy level (455 nm) through cross-relaxation. The imaging resolution of a single NaYF_4_:Yb,Tm UCNP, which has a physical size of 13 nm, reached *ca*. 28 nm. Almost simultaneously, Zhan et al. reported the STED imaging with heavily doped NaYF_4_:Yb,Tm UCNPs (Zhan et al., 2017). Importantly, the authors realized blue (Tm^3+^) and green (Tb^3+^) bi-color STED imaging with core/shell structured NaGdF_4_:Yb,Tm@NaGdF_4_:Tb UCNPs. Moreover, STED imaging of cytoskeleton structures and desmin proteins in HeLa cells was realized with antibody modification, and the optimal resolution was down to 82 nm (Figure 1E). A recent study shows that the doping ratio of Tm^3+^ in NaYF_4_:Yb,Tm can be increased from 8 to 20% with no influence on the STED imaging performance (Camillis et al., 2020). Meanwhile, the content of Yb^3+^ can be enriched to *ca*. 90% by forming NaYbF_4_:Tm. The prominent increase in Yb^3+^ content also benefits to accelerate emission kinetics of Tm^3+^, which enables fast STED imaging capability. The pixel dwell time for the STED imaging of NaYbF_4_:Tm UCNPs can be shortened from a few milliseconds to 10 μs (Peng et al., 2019), which is close to that for organic dyes. The simultaneous improvement in spatial and temporal resolution benefits to visualize the dynamics of cells and tissues.

Till now, UCNP-based probes for STED microscopy still concentrate on Yb^3+^-Tm^3+^-activated nanoparticles, suggesting the difficulty in modulating the upconversion emission properties of lanthanide ions under the STED mode. In addition, the blue emissions of Tm^3+^ still face the obstacle from limited penetration and potential photodamage; thus, exploring novel UCNP-based STED probes with longer working wavelength is desirable.

### UCNPs in Fluorescence Emission Difference Microscopy

FED microscopy relies on the subtraction of two distinct images taken under excitations of a solid laser and a doughnut-shaped laser, respectively (Figure 1F) (Kuang et al., 2013). The super-resolved FED image is obtained by setting the negative values to zero with the equation *I*_FED_ = *I*_solid_ – *r* × *I*_doughnut_, in which the *I*_FED_, *I*_solid_, *I*_doughnut_, and *r* represent the normalized FED intensity profile, solid laser scanning profile, doughnut-shaped laser scanning profile, and subtractive factor, respectively. The more intense the doughnut-shaped laser is, the better the imaging resolution will be. In principle, there is no specific requirement for the composition of UCNPs. Nonetheless, because of the requirement of image processing, FED is not advantageous in temporal resolution. In 2017, Zhan et al. described the first UCNP-based FED microscopy (Figure 1G) (Wu et al., 2017). NaYF_4_:Nd,Yb,Er@NaYF_4_:Nd UCNPs were excited by 808-nm solid and doughnut-shaped lasers, respectively, with a power density of 10^7^ W/cm^2^. The resolution was improved from 440 to 170 nm for single UCNPs. In a typical FED imaging, two separate scans are required to acquire the solid and doughnut-shaped images. Recently, Zhan et al. achieved one-scan FED microscopy with NaYF_4_:Er@NaYF_4_@NaYF_4_:Yb,Tm UCNPs (Huang et al., 2018). The UCNPs show blue emissions of Tm^3+^ under 940-nm solid laser excitation while green emissions of Er^3+^ under 808-nm doughnut-shaped laser excitation. With the orthogonally luminescent UCNPs, a resolution of 54 nm was obtained. Aiming at achieving deep penetration, Jin et al. investigated the FED imaging on the basis of the NIR emission of NaYF_4_:Yb,Tm UCNPs (Chen et al., 2018). They obtained a resolution of sub-50 nm in imaging single UCNPs through 93-μm-thick liver tissue.

### UCNPs in Structured Illumination Microscopy

SIM refers to the wide-field illumination of samples with light that has closely packed periodic patterns (Gustafsson, 2000). Upon interference with samples, Moiré patterns that contain high spatial frequency information of samples are obtained, from which the super-resolved image can be reconstructed through deconvolution algorithm. However, the strong extinction of light by biological tissues, which are deleterious to imaging depth and resolution, restricts further bioapplications of SIM microscopy. This problem can be conquered by using UCNPs as the SIM probes. Very recently, Jin et al. employed NaYF_4_:Yb,Tm UCNPs as SIM probes and achieved a resolution below 131 nm and an imaging rate of 1 Hz (Figure 1H) (Liu et al., 2020). The authors show that the non-linear photoresponses of UCNPs can produce high-frequency harmonics in the Fourier domain of the imaging plane, enabling non-linear SIM under mild excitation conditions (10^3^ W/cm^2^). Moreover, Tm^3+^-activated NIR-emissive UCNPs are found superior to visible-emissive counterparts in preserving the structured spatial information through thick tissue. It is noteworthy that the non-linear SIM can generate improved resolution compared to the linear SIM; thus, the doping ratio of lanthanide ions, which can influence both the non-linearity and intensity of upconversion emissions, should be carefully optimized.

## Perspectives of UCNPs-Based Super-Resolution Imaging

### Small-Sized UCNPs With Bright Emissions

**Figure 2:**
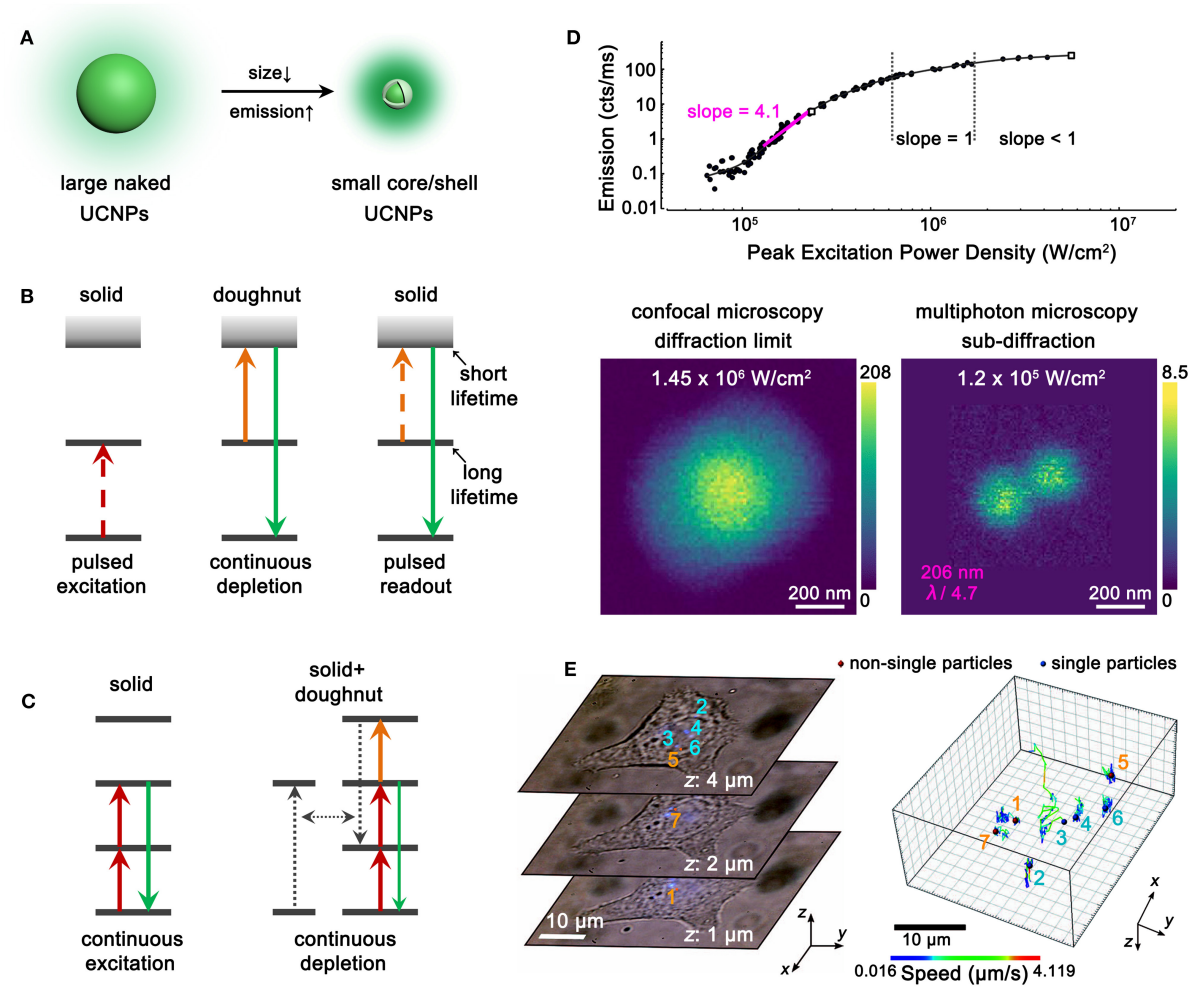
Perspectives of UCNPs-based Super-Resolution Imaging. **(A)** Schematic illustration showing the requirement of size decrease and emission enhancement with core/shell UCNPs. **(B)** Schematic illustration showing the UCNPs with long intermediate-level lifetime and short excited-state lifetime for STED microscopy. **(C)** Schematic illustration showing the combination of excitation and non-radiative cross-relaxation in UCNPs for STED microscopy. **(D)** Non-linear photoresponses of NaYF_4_:Yb,Tm UCNPs at 455 nm under 980-nm excitation (top) and comparative imaging of two adjacent UCNPs under 980-nm excitation with different power densities (bottom). Reproduced with permission from Denkova et al. (2019). Copyright 2019 Springer Nature Publishing Group. **(E)** Three representative single-cell images containing seven UCNP spots (left) and the 3D trajectories of the UCNPs (right). Reproduced with permission from Wang et al. (2018). Copyright 2018 Springer Nature Publishing Group.

The parity-forbidden nature of 4*f* intra-configurational transitions makes lanthanide upconversion a low-efficiency process. Deleterious coupling with surface defects, ligands, and solvent molecules would further aggravate the upconversion brightness (Dong et al., 2015b). To obtain adequate luminescence from single UCNPs, large-sized nanoparticles (larger than 20 nm) and high pump power (more than 10^4^ W/cm^2^) are usually required in super-resolution microscopy. However, both the size effect (Albanese et al., 2012) of UCNPs and the heating effect (Peña et al., 2012) of high pump power may interfere with the intrinsic activities of as-labeled biomolecules or the whole cells, leading to misapprehensive observations. Moreover, the imaging resolution is limited by the physical size of UCNPs to some extent; thus, the ultimate resolution is larger than 20 nm in current studies. To improve the application performance of UCNPs and avoid interference with cellular activities, small-sized UCNPs with bright emissions should be developed. It has been reported that sub-5 nm NaLnF_4_ UCNPs can be obtained with co-precipitation synthesis (Xing et al., 2014; Zheng et al., 2017), and an epitaxial shell can greatly enhance the upconversion emissions of core nanoparticles by orders of magnitudes (Dong et al., 2017). Therefore, sub-10-nm bright UCNPs are expected to be prepared with the sub-5-nm core and an epitaxial shell (Figure 2A). The decrease in nanoparticle size should facilitate both the efficient labeling of biomolecules and improved imaging resolution.

### Tailoring Upconversion for Novel Super-Resolution Probes

As discussed above, the as-developed UCNPs probes for super-resolution microscopy are mainly focused on Yb^3+^-Tm^3+^ co-doped nanoparticles. Generally, there are no rigid restrictions on the types of luminescent centers for ion beam excitation, FED and SIM. UCNPs doped with other lanthanide ions such as Er^3+^, Ho^3+^, Nd^3+^, etc. can be examined. In comparison, STED microscopy strictly requires depleted upconversion emission under co-illumination of excitation and depletion lasers, which is indeed challenging for lanthanide ions with ladder-like energy levels. Adequate cross-relaxation pairs that can depopulate the emissive levels under STED mode should be explored. It can be referred to the example that takes advantage of lifetime difference in YAG:Pr UCNPs (Kolesov et al., 2011), in which long and short lifetimes are preferred for the intermediate and excited states, respectively (Figure 2B). Moreover, the combination of excitation and non-radiative relaxation/cross-relaxation can be considered as a new depletion approach for STED microscopy (Figure 2C) (Wu et al., 2015). Besides, probes for photoactivated localization microscopy (PALM) (Betzig et al., 2006), stochastic optical reconstruction microscopy (STORM) (Rust et al., 2006), and super-resolution optical fluctuation imaging (SOFI) (Dertinger et al., 2009) are desired to be explored with the composites of UCNPs and fluorescent proteins, organic dyes, and quantum dots, respectively. Meanwhile, fast decay rates of UCNPs are preferred for real-time imaging with high temporal resolution (Peng et al., 2019). Thus, rational screening of the host matrices, doping types, and concentrations of lanthanide ions should also be considered for exploring novel UCNP probes for super-resolution microscopy.

### Exploiting High-Order Non-linearity of UCNPs for High-/Super-Resolution Microscopy

Except for the canonical super-resolution techniques, multiphoton probes with high order of non-linearity favors higher spatial resolution (Yu et al., 2013; Bednarkiewicz et al., 2019). In multiphoton imaging, the imaging resolution approximately equals to λ/(2 × √*N*), where λ and *N* denote the excitation wavelength and the order of non-linearity, respectively. Importantly, the high-resolved image can be directly achieved on a laser-scanning confocal microscopy without the requirements of complex systems and image post-processing. This is ascribed to the fact that only the central and most intense region of the excitation beam can induce adequate emissions from multiphoton probes, while the periphery of the beam barely excites the probes. To some extent, imaging with multiphoton probes also alleviates the requirement of the pinhole because of less interference from out-of-focus light. Therefore, exploring UCNPs with highly non-linear photoresponses are greatly desirable. The four-photon 455-nm emission of Tm^3+^ is a good paradigm for achieving high-resolution imaging. Denkova (Denkova et al., 2019) and Zvyagin (Kostyuk et al., 2019) et al. realized a *ca*. 200-nm resolution in heavily doped NaYF_4_:Yb,Tm UCNPs under 980-nm excitation (Figure 2D). Zhan and coworkers improved the resolution to 161 nm by blue-shifting the excitation wavelength to 730 nm with Nd^3+^/Yb^3+^/Tm^3+^ tri-doped UCNPs (Wang et al., 2016). UCNPs with higher non-linearity and longer emission wavelength are preferred to achieve higher/super resolution and improved tissue penetration. Very recently, Schuck et al. developed Tm^3+^-activated photon avalanching UCNPs with giant non-linear photoresponses (*N* up to 26) for the 800-nm NIR emissions and achieved a resolution of *ca*. 70 nm (Lee et al., 2020). With the unique advantage of rich energy levels, more intriguing multiphoton upconversion processes in lanthanide-doped UCNPs are desired for high-/super-resolution microscopy.

### Applying UCNPs for Cellular Biology

The super-resolved cellular biology application of UCNPs is still in the preliminary stage compared with that of conventional probes (Jin et al., 2018). Great efforts should be devoted to resolve the morphological and physiological characters of living cells, subcellular organelles, proteins, etc., such as the structure and communications of neuron cells (Xu et al., 2013; Tang et al., 2016) as well as the translational and rotational movements of motor proteins (Ohmachi et al., 2012). Labeling of targeted biomolecules precisely requires solid foundation on the surface modification of UCNPs; thus, the effective loading of DNA strands, peptides, antibodies, etc. that have targeting ability should be addressed.

Each super-resolution microscopy has its unique advantages and limitations, yet the combination of the techniques might generate further improved performance. For example, the implantation of multiphoton probes with large non-linearity into STED/FED/SIM should result in higher resolution. The conjunction of SIM and STED should enable video-rate and large-view super-resolution imaging. In this way, the interactions between UCNPs and cells can be disclosed in nanoscale accuracy, including the internalization and externalization of UCNPs by cells as well as the subcellular trajectories of UCNPs (Figure 2E). The size-, morphology-, and surface charge-dependent interactions can also be studied. Moreover, the subcellular microenvironments including the viscosity (Wang et al., 2018) and temperature (Rodríguez-Sevilla et al., 2016) can be unraveled by the super-resolved upconversion images with UCNPs.

## Discussion

Lanthanide-doped UCNPs are being developed as probes for diverse super-resolution microscopy. The excellent photostability, adequate multiphoton emission efficiency, and tunable emission colors and lifetimes make UCNPs stand out among various as-developed super-resolution probes, especially in long-term and real-time imaging applications. Nonetheless, the complex energy levels and the small stimulated emission cross-section of lanthanide ions are still considerable challenges for flexible design of UCNPs for super-resolution techniques such as STED microscopy. Moreover, the particle size should be reduced and the heating effects should be avoided by exploring small-sized and bright UCNPs. With the development of UCNP probes, the super-resolved cellular biology applications of UCNPs are ready to be implemented. There is still plenty of room for UCNP-based super-resolution microscopy, and it is believed that UCNPs would attract great concern as probes for super-resolution microscopy in the near future.

## Data Availability Statement

The original contributions presented in the study are included in the article/supplementary material, further inquiries can be directed to the corresponding author.

## Author Contributions

All authors listed have made a substantial, direct and intellectual contribution to the work, and approved it for publication.

## Conflict of Interest

The authors declare that the research was conducted in the absence of any commercial or financial relationships that could be construed as a potential conflict of interest.
